# Virion Secretion of Hepatitis B Virus Naturally Occurring Core Antigen Variants

**DOI:** 10.3390/cells10010043

**Published:** 2020-12-30

**Authors:** Chiaho Shih, Szu-Yao Wu, Shu-Fan Chou, Ta-Tung Thomas Yuan

**Affiliations:** 1 Graduate Institute of Medicine, Kaohsiung Medical University, Kaohsiung 80708, Taiwan; 2 Institute of Biomedical Sciences, Academia Sinica, Taipei 11529, Taiwan; 3 Chimera Bioscience Inc., No. 18 Siyuan St., Zhongzheng Dist., Taipei 10087, Taiwan; guguma126@yahoo.com.tw; 4 Department of Microbiology, Harvard Medical School, Boston, MA 02115, USA; fanze2025@yahoo.com.tw; 5 TFBS Bioscience, Inc. 3F, No. 103, Ln 169, Kangning St., Xizhi Dist., New Taipei City 221, Taiwan

**Keywords:** hepatitis B virus (HBV), naturally occurring mutation, HBV core antigen, immature virion secretion, genome maturation, hydrophobic pocket, compensatory mutation, persistence, hydrodynamic mouse model

## Abstract

In natural infection, hepatitis B virus (HBV) core protein (HBc) accumulates frequent mutations. The most frequent HBc variant in chronic hepatitis B patients is mutant 97L, changing from an isoleucine or phenylalanine to a leucine (L) at HBc amino acid 97. One dogma in the HBV research field is that wild type HBV secretes predominantly virions containing mature double-stranded DNA genomes. Immature genomes, containing single-stranded RNA or DNA, do not get efficiently secreted until reaching genome maturity. Interestingly, HBc variant 97L does not follow this dogma in virion secretion. Instead, it exhibits an immature secretion phenotype, which preferentially secretes virions containing immature genomes. Other aberrant behaviors in virion secretion were also observed in different naturally occurring HBc variants. A hydrophobic pocket around amino acid 97 was identified by bioinformatics, genetic analysis, and cryo-EM. We postulated that this hydrophobic pocket could mediate the transduction of the genome maturation signal for envelopment from the capsid interior to its surface. Virion morphogenesis must involve interactions between HBc, envelope proteins (HBsAg) and host factors, such as components of ESCRT (endosomal sorting complex required for transport). Immature secretion can be offset by compensatory mutations, occurring at other positions in HBc or HBsAg. Recently, we demonstrated in mice that the persistence of intrahepatic HBV DNA is related to virion secretion regulated by HBV genome maturity. HBV virion secretion could be an antiviral drug target.

## 1. Introduction

**Figure 1:**
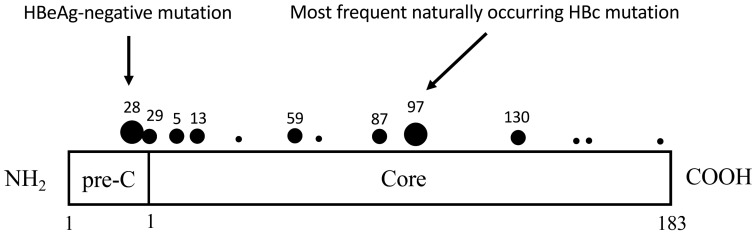
Coincidence of naturally occurring HBc mutations with mapped T cell epitopes in literature. Large dots, more than 45%; medium dots, 20% to 35%; and small dots, fewer than 20% of HBV-infected patients are found to have virus with a predominant mutation at this position (adapted with permission from Hosono et al., 1995) [12].

Hepatitis B virus (HBV) is a major human pathogen [1,2]. Chronic infection with HBV could lead to chronic hepatitis, cirrhosis and hepatoma [3,4]. At present, no curative treatment can effectively eradicate the virus from patients [5,6,7]. HBV replicates via a sloppy polymerase containing a reverse transcriptase domain with low fidelity in DNA synthesis. Therefore, HBV in chronic carriers exists as a quasispecies, rather than one single homogeneous population. The most frequent naturally occurring mutation in HBV core protein (HBc) occurs at amino acid 97, changing an isoleucine or phenylalanine to leucine (I97L or F97L). This hotspot mutation was found in HBV DNA in the sera of Japanese hepatitis B patients with liver injury, or severe fulminant hepatitis [8,9,10], as well as in the integrated HBV DNA in Taiwanese hepatoma samples (Figure 1) [11,12]. In asymptomatic carriers and self-limited acute hepatitis B, this mutation was not found. Furthermore, this HBc 97L mutation is present not only in Asian patients but also in Italians with chronic infection [13]. Therefore, 97L mutation can be found in different ethnic groups in different areas of the world. 

What could be the functional significance of this HBc mutation at amino acid 97? Hosono et al. [12] reported that HBc mutations at codons 5, 97 and 130, all coincide with HLA class II-restricted T cell epitopes. In the woodchuck model infected with woodchuck hepatitis virus (WHV), peptides 1–20, 97–110, and 112–131 were shown to be major T-cell epitopes of woodchuck hepatitis B core antigen [14]. Similarly, peptide 91–105, which overlaps with amino acid 97, produced maximal proliferation of the peripheral blood lymphocytes from chronic WHV carriers [15]. Although naturally occurring HBc mutation could fall within the target epitopes of CTL (cytotoxic T lymphocytes) in human patients, more direct and rigorous evidence for this immune escape interpretation remains to be further examined in animal models.

## 2. An Immature Secretion Phenotype of Mutant 97L 

**Figure 2:**
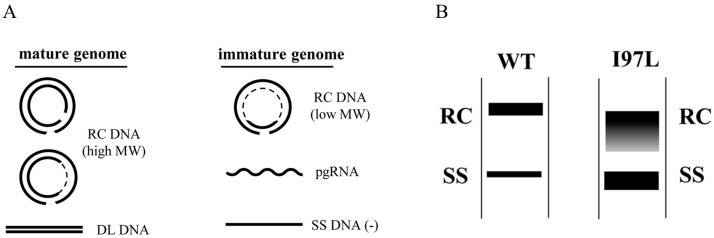
HBV core variants 97L exhibited abnormal behaviors in viral DNA synthesis and virion secretion. (**A**) Cartoon illustrations of mature and immature HBV genomes. pgRNA: pregenomic RNA; SS DNA: single-strand (−) DNA reversed transcribed from pgRNA (+); RC DNA: partially double-strand relaxed circle DNA. DL: Low abundant double-strand linear DNA can be separated from RC DNA after longer time electrophoresis. A dotted line of the RC molecules represents the single-strand gap region with a variable size in an HBV population. (**B**) Different Southern blot patterns of virion-associated HBV DNA genomes from wild type and HBc mutant I97L (with permission from Wu et al., 2019) [23].

Beyond its potential significance in immunity, mutant 97L displays pleiotropic phenotypes. In particular, one dogma in the field is that, for a wild type hepadnavirus, only virions containing a mature genome (Figure 2A) are preferentially secreted into the medium [16]. This dogma is violated by mutant 97L. For wild type HBV in cell culture, the ratio between the secreted mature genome and immature genome is generally around 4 to 1. For this variant 97L, it secretes almost equal amounts (1:1) of mature and immature genomes. Therefore, one major characteristic feature of mutant 97L is its secretion of excessive amount of virions containing immature genomes (Figure 2B). In other words, mutant I97L lost the high stringency of selectivity in genome maturity during virion export. This is a widely observed phenotype since it is true in HBV subtypes *ayw* [17], *adr* [18] and genotype A [19]. In addition, immature secretion is an evolutionarily conserved phenomenon found in other non-human hepadnaviruses, such as snow goose hepatitis B virus (SGHBV) [20,21]. In a woodchuck hepatitis B virus (WHV) model, when WHV-infected woodchucks were treated with acyclovir, minus-strand viral DNA were enveloped and secreted as virion-like particles [22]. 

To investigate the mechanism of immature secretion, we compared the kinetics of virion secretion between the immature and the mature genomes in mutant F97L as well as the secretion of mature genomes between the WT and the mutant F97L. In the case of variant F97L, the immature genome was detectable on day 3, whereas the mature genome appeared on day 2 post-transfection. Furthermore, there is no kinetic difference in the secretion of mature genomes between WT HBV and mutant F97L, both being detectable on day 2 post-transfection by Southern blot analysis. In summary, the variant F97L does not secrete its mature or immature virions any earlier or faster than the WT HBV [24].

## 3. A Cis-Trans Genetic Complementation Test

**Figure 3:**
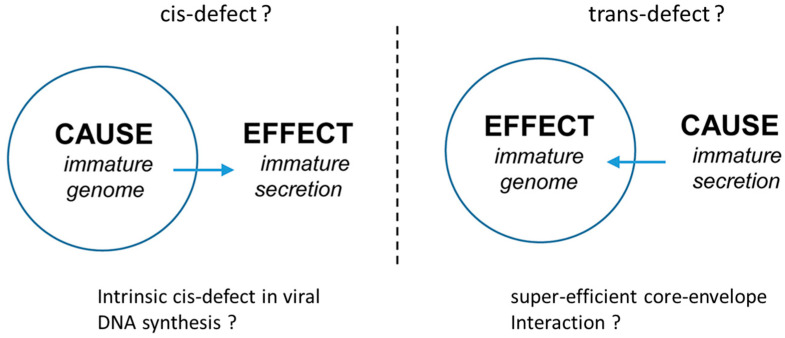
A cartoon illustration in dissecting two opposite cause-effect relationships between genome maturation and virion secretion of HBc mutant 97L (with permission from Wu et al., 2019) [23].

**Figure 4:**
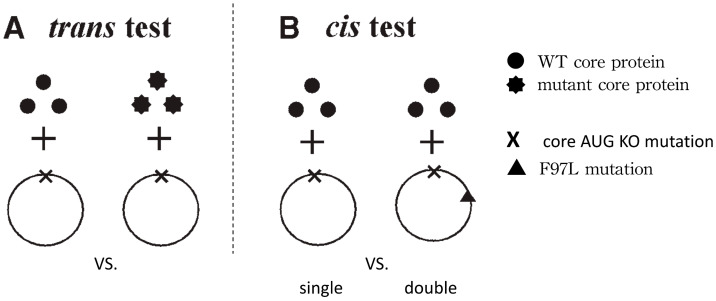
A cis-trans complementation test is designed to investigate the cause-effect relationship between the extracellular phenotype of virion secretion and the intracellular phenotype of viral DNA synthesis. HuH-7 cells were cotransfected by using two different plasmids as shown in the figure. Viral DNA synthesis can be assayed by Southern blot analysis. (**A**) trans-test. Two different core proteins, wild-type (smooth dots) vs. mutant (starry dots), were compared side by side for their respective abilities to rescue the same core AUG knockout (KO) mutant 1903. (**B**) cis-test. The same wild-type core protein was used to rescue two different replication-defective HBV: the core-defective single-mutant 1903 (X symbol) vs. the double-mutant 1903/I97L containing an additional I97L mutation (triangle symbol). (with permission from Shih et al., Methods Mol. Med., 2004) [25].

Using strand-specific probes in Southern blot analysis, Yuan et al. [17] demonstrated that one intracellular phenotype of mutant F97L is the decrease in the overall HBV DNA replication, particularly in the plus-strand DNA synthesis. It is unclear if this deficiency in viral DNA synthesis is due to a trans-defect in the mutant F97L core protein, or a cis-defect in the mutant HBV genome per se. Nor is it clear if the extracellular phenotype of immature virion secretion could originate from any aberrant or super-efficient core-envelope interaction in virion morphogenesis, which in turn diminishes the intracellular HBV DNA pool (Figure 3). For example, the primary cause of the intracellular HBV DNA deficiency could result from an intrinsic defect in intracellular HBV DNA synthesis and genome maturation, leading to the extracellular immature virion secretion. To dissect these tightly intertwined chicken-and-egg issues, we designed a cis-trans genetic test (Figure 4) [25].

**Figure 5:**
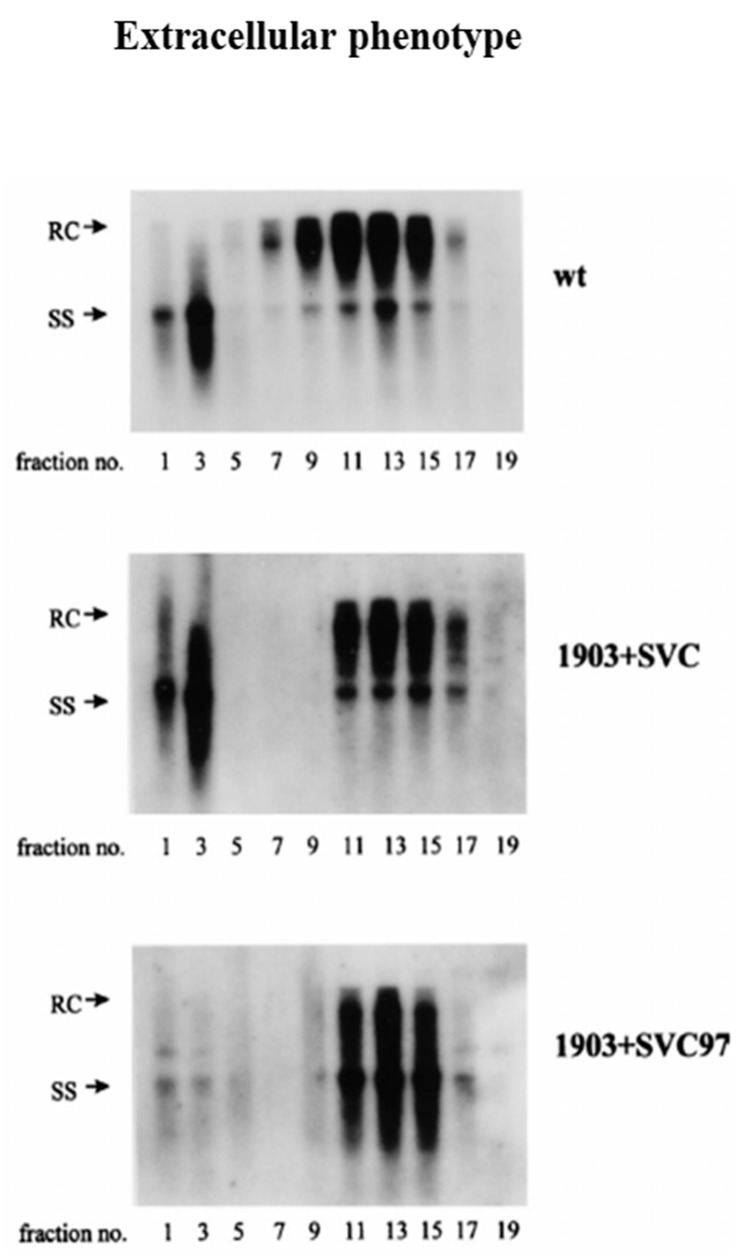
A complementation assay revealed the trans-defect of mutant F97L core protein in the extracellular phenotype of immature virion secretion. Co-transfection of the core-deficient mutant 1903 with the mutant F97L is necessary and sufficient to reproduce the viral DNA pattern of immature virion secretion. Media from the transfected culture were fractionated through density gradient centrifugation. HBV DNA signals peaked around fraction # 11–15 by Southern blot analysis. 1903: core-deficient mutant, SVC: WT-HBc expression vector, SVC97: F97L-HBc expression vector. (with permission from Yuan et al., 1999) [17].

**Figure 6:**
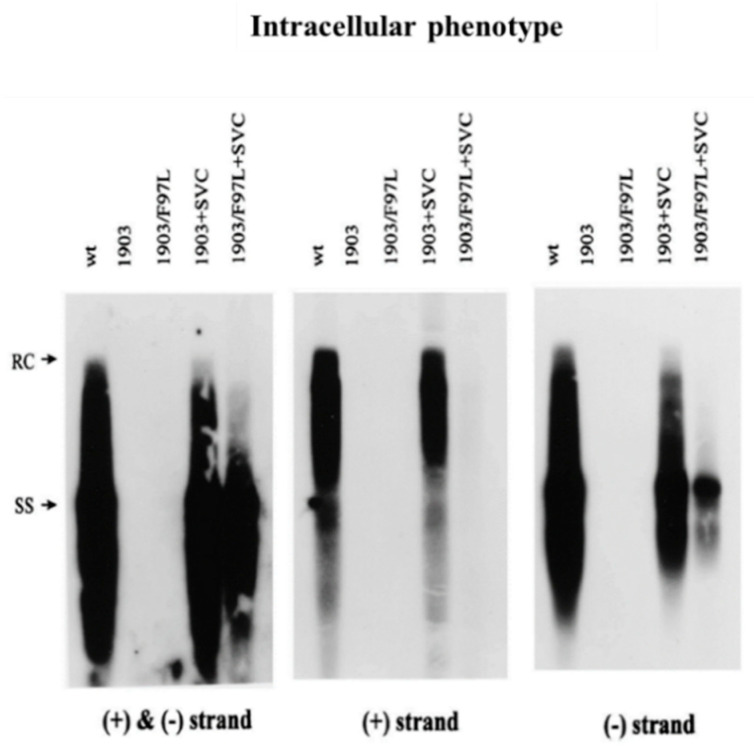
A complementation assay revealed the cis-defect of mutant F97L in the intracellular deficiency in both plus-strand and minus-strand DNA syntheses. As illustrated in Figure 4, a wild-type (wt) core protein expression vector was cotransfected with either single mutant 1903 or double mutant 1903/F97L into HuH-7 cells. Strand-specific probes were used for Southern blot analysis of intracellular core-associated DNA. (with permission from Yuan et al., 1999) [17].

Our cis-trans complementation test (Figure 4) demonstrated that mutant F97L exhibited both a trans-defect of the mutant HBc protein in the extracellular immature virion secretion (Figure 5), as well as a cis-defect of a mutant genome in the intracellular viral DNA synthesis (Figure 6). 

**Figure 7:**
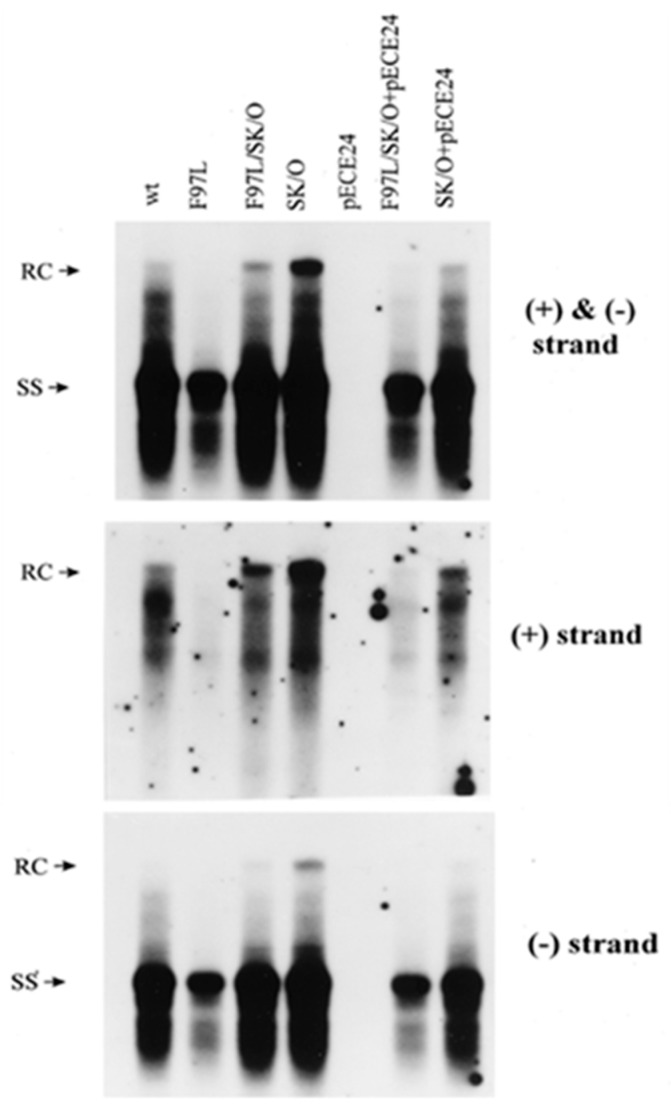
Dissociation of a cis-defect from a trans-defect by blocking HBsAg synthesis and virion secretion of HBc mutant 97L. Co-transfection experiments were conducted as described in Figure 4 legend. The intracellular plus-strand DNA deficiency of mutant F97L is mainly caused by a cis-defect when virion secretion is blocked by abrogating the initiation codon of HBsAg (middle panel; compare lanes F97L/SK/O and SK/O). The blockage of virion secretion can be rescued by supplying HBsAg from a cotransfected expression vector pECE24. The intracellular minus-strand DNA deficiency of mutant F97L is most apparent when the mutant F97L virus can be secreted in the presence of HBsAg and a mutant F97L HBc (lower panel; compare the last two with the middle two lanes). (with permission from Yuan et al., 1999) [17].

In addition to the cis-trans test, we designed another experiment to probe the existence of a cis-defect of this clinically prevalent mutant F97L (Figure 7). HBsAg surface antigen is known to be required for HBV virion secretion [26]. We inhibited virion secretion of both WT-HBV and mutant F97L by abrogating their HBsAg protein synthesis (SKO). If immature virion secretion is the primary cause of the intracellular viral DNA deficiency, one should expect to rescue the intracellular DNA deficiency by blocking the virion secretion of mutant F97L. The results in Figure 7 indicate that intracellular DNA deficiency of mutant F97L can only be partially rescued (30%) by blocking the initiation codon of HBsAg and virion secretion (Figure 7, compare lanes F97L/SK/O and SK/O). In other words, around 70% of the intracellular DNA deficiency could be attributed to a cis-defect from the mutant F97L genome. Taken together, results from Figure 5 indicated that the mutant F97L HBc protein is mainly responsible for the extracellular phenotype of immature virion secretion. Moreover, results from Figure 6 and Figure 7 indicated that the intracellular plus-strand DNA deficiency is at least in part caused directly by a cis-defect of the mutant genome. Plus-strand DNA synthesis is known to use the minus-strand DNA template during reverse transcription. Therefore, part of the intracellular plus-strand DNA deficiency must be contributed indirectly from the immature virion secretion, which preferentially drains off the intracellular minus-strand DNA to the extracellular compartment. Finally, the intracellular minus-strand DNA deficiency of mutant F97L appeared to be caused by a cis-defect of a mutant genome in DNA synthesis, as well as by a trans-defect of a mutant HBc, which depleted the intracellular immature genome (minus-strand DNA) via immature virion secretion.

## 4. A Hydrophobic Pocket around Amino Acid 97 in Signal Transduction

**Figure 8:**
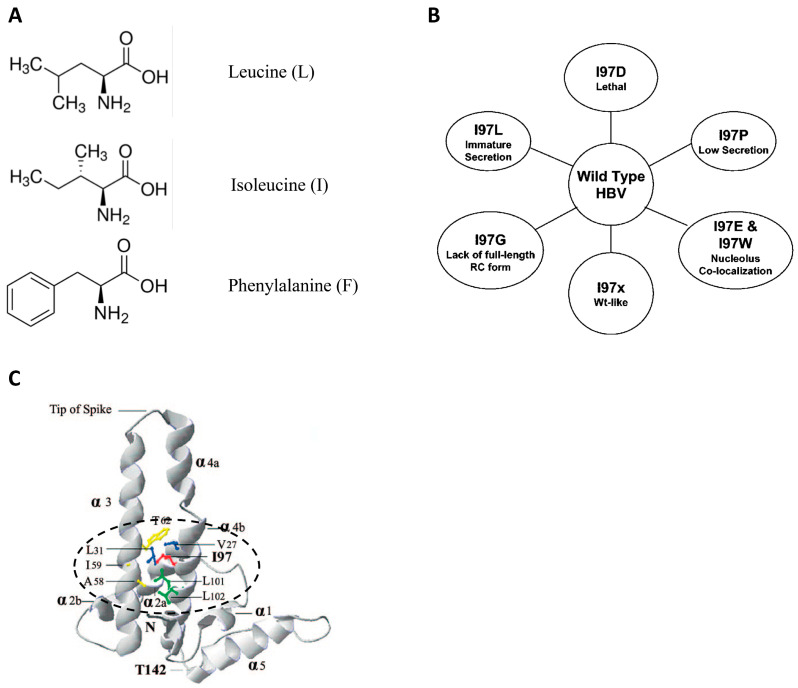
Dissecting the structure-function relationship of mutant 97L-associated phenotypes. (**A**) Structural similarity between hydrophobic amino acids leucine, isoleucine, and phenylalanine. (**B**) Only mutation I97L can generate the immature secretion phenotype. (**C**) A hydrophobic pocket (circled by a dotted line) could be identified by bioinformatics within 7-angstrom distance from the isoleucine at amino acid 97 of a monomer HBc. An atomic structure of HBc icosahedral particles is based on the cryoEM and X-ray diffraction analyses [27,28,29,30] (adapted with permission from Ning and Shih, 2004) [31].

Hydrophobic amino acids isoleucine and leucine are structural isomers with the same molecular weight (Figure 8A). It is intriguing that such a subtle structural difference in their side chains could result in a drastic phenotype of immature secretion. To elucidate the structure-function relationship of the pleiotropic phenotypes associated with mutant 97L, we systematically replaced the isoleucine residue at this position with 18 other amino acids via mutagenesis. Our results from various functional assays indicated that it is the specific acquisition of a leucine residue at HBc amino acid 97, rather than the loss of an isoleucine, that is necessary and sufficient for the immature secretion phenotype (Figure 8B). Five engineered mutants displayed various new phenotypes. Interestingly, based on the known atomic structure of the assembled icosahedral HBc particles [27,28,29,30], a hydrophobic pocket around amino acid 97 (Figure 8C) was identified by bioinformatics [31] and cryoEM [32,33]. This hydrophobic pocket could be involved in the relay of the genome maturation signal from the capsid interior to the surface of capsids undergoing genome maturation. For example, it is known that a dominant conformational epitope is present at the tip of the spike (aa 78–82), which can be recognized by an anti-HBc antibody [34]. An I97E mutation at the hydrophobic pocket significantly attenuated this dominant epitope at the tip of the spike [31]. Similarly, histidine tagging at the C terminus of several different mutant HBc in the capsid interior also resulted in the complete loss of this distal dominant epitope at the tip of the spike [31]. Taken together, we hypothesized here that the propagation of the signal of genome maturation from the capsid interior to the capsid surface, must go through this hydrophobic pocket. A mutant 97L hydrophobic pocket is somehow faulty in maintaining the tight coupling of the tempos between genome maturation and virion secretion.

Empty virions had been observed in patients and cell culture [19,35,36,37,38,39]. Since empty virions are genome-free, their virion release should be independent of genome maturation. A single-strand blocking model was proposed to accommodate the virion secretion of both genome-free and genome-containing virions [39]. It proposed that encapsidated single-strand (SS) nucleic acids could serve as an inhibitory signal for envelopment and virion secretion. One important unknown here is whether both genome-free and genome-containing virions are really secreted by the same route. Neither the classic genome maturation model [16] nor the revised single-strand blocking model, are consistent with the immature secretion of virions containing single-strand DNA [17,18,19,20,21,22,23,24,25]. The temporal relationship between genome maturation and virion secretion deserves further investigation in the future.

## 5. Capsid Stability, Assembly, and Morphology of HBc I97L

To better understand the structural basis of immature secretion, we compared the stability and morphology between WT- and mutant-97L capsid particles, in either full-length (HBc 1-183) or truncated core protein contexts (HBc 1-149 and HBc 1-140). We found no significant differences in capsid stability between wild-type and mutant I97L particles under different stress conditions, such as denaturing pH, temperature, and SDS treatments [40]. Similar results were reported recently by comparing the stability of E. coli-expressed capsid-like particles (CLP) against SDS between wild type HBc and mutant F97L [41]. HBV capsids are known to exist in natural infection as dimorphic T = 3 or T = 4 icosahedral particles. In the context of HBc1-140, we compared wild-type HBV and mutant I97L and found similar ratios between T = 3 (78%) and T = 4 particles (20.3%). In addition, we observed no difference in capsid stability between T = 3 and T = 4 particles. Although no apparent difference in capsid morphology and stability can be detected between WT and mutant F97L, the latter appeared to exhibit enhanced rate and extent of capsid assembly [42].

## 6. Compensatory Mutations for the Immature Secretion

### 6.1. Low Virion Secretion and Compensatory Mutations

In addition to mutation I97L, we identified two novel and frequent HBc mutations P5T and P130T in Taiwanese hepatomas (Figure 1) [12]. A single mutation P130T displayed an intracellular “genome hypermaturation” phenotype with excessive accumulation of near full-length RC DNA [43]. The nonselective immature secretion of mutant F97L can be efficiently offset by mutation P130T, in the context of the double mutant F97L/P130T [43]. Similarly, mutation P5T is compensatory in offsetting the immature secretion of I97L. Mutation P5T, by itself, can generate a low level of virion secretion [44]. Such a phenotype of low virion secretion was also observed in a number of artificially engineered HBc mutants [45]. None of these engineered HBc mutants has ever been found to mimic an immature secretion phenotype like the naturally occurring mutant HBc 97L. 

Previously, an engineered mutant HBc 164, which has a C-terminal truncation at HBc 165–183, was once thought to secrete immature genomes [46]. Further studies on the immature secretion-like phenotype of HBc mutant 164, revealed that the capsid-associated low MW DNAs are not bona fide single-stranded (minus-strand) immature genomes. Heat denaturation experiment and a plus-strand-specific probe by Southern blot demonstrated that the intracellular DNA of mutant 164 capsids contained shorter single-stranded and double-stranded DNA replicative intermediates [47,48]. Mutations at the major splice sites (nt 487 or/and nt 2449) eliminated strong DNA signals of mutant 164 [48]. In sum, the engineered mutant 164 does not have the same immature secretion phenotype as the naturally occurring mutant 97L. In fact, HBV capsids and virions could contain many different species of double-stranded spliced DNA reverse transcribed from encapsidated spliced RNAs [49].

By serendipity, in a clinical isolate from Shanghai, which contains both I97L and P5T, we found neither immature secretion nor low virion secretion. Further studies revealed that P5T and I97L were mutually compensatory for each other [50]. In fact, an effort to identify the immature secretion phenotype in patients is more often complicated by several factors: (1) Most patient samples contain a mixture of both WT and mutant 97L at different ratios [51]. (2) Known or unknown compensatory mutations could offset the immature secretion phenotype [43,50]. (3) The low MW HBV DNA in the blood samples could result from either DNA degradation during sample storage or preparation; or result from (4) the encapsidation and reverse transcription of spliced viral RNAs [47,48,49,52,53], rather than from the bona fide immature viral genomes.

### 6.2. PreS1 LHBs Compensatory Mutation

Virion morphogenesis involves HBV core-envelope interactions. We hypothesized that the immature secretion phenotype could be caused by the aberrant intermolecular interaction between its mutant core and wild-type envelope proteins. If so, is it possible that some of the naturally occurring mutations of HBsAg, in addition to the aforementioned HBc P5T or P130T, could be compensatory for the 97L immature secretion? Previously, we identified frequent naturally occurring point mutations of HBsAg in Taiwanese hepatomas, including mutations at the small surface antigen hotspot codons 40 and 47, group a determinant, [54], as well as preS2 immune escape deletions [55]. A common phenotype of HBsAg deletion or point mutation is the accumulation of mutant HBsAg in the ER-Golgi, resulting in ER stress [51,56]. Pre-S2 deletion protein has also been associated with occurrence or recurrence of HBV-related hepatomas [57,58,59,60]. Can any of these naturally occurring HBsAg mutations have any compensatory effect on the rescue of an immature secretion phenotype? 

While we had not so far identified any HBsAg compensatory mutations for the immature secretion of HBc 97L, we took another approach by site-directed mutagenesis to create potential compensatory HBsAg mutations [24]. After screening a number of pre-S1 mutants, we identified a pre-S1 large envelope (LHBs) mutation at position 119, changing an alanine (A) to a phenylalanine (F). By itself, this engineered mutation preS1-A119F (not a naturally occurring mutation) exhibited a low-secretion phenotype. However, it can offset the immature secretion phenotype of the mutant I97L, and successfully restore the WT-like selective export of the mature genome of the double mutant preS1-A119F/HBc-I97L.

## 7. Host Factors in Virion Secretion

As a hepatotropic DNA virus, HBV replication can be strongly influenced by transcription factors or other liver specific factors. In this section, we will focus on host factors in virion secretion. 

### 7.1. Vps4 ATPase 

**Figure 9:**
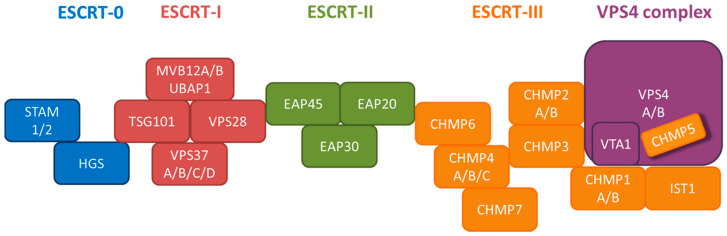
A cartoon illustrates the ESCRT cascade and the interactions between each component members of ESCRT-0, I, II, III, and the VPS4 ATPase complex. Of note, the exact stoichiometry of certain component members remains tentative (with permission from Chou et al., 2015) [61].

The endosomal sorting complex required for transport (ESCRT) is a house-keeping intracellular machinery for both sorting and trafficking of ubiquitinated protein cargos [61] (Figure 9). Vps4 is another host factor known to be involved in cellular vacuolar protein sorting. Although Vps4 is not an integral part of the ESCRT complex, its activity is often associated with the ESCRT-mediated membrane dynamics. We and others reported independently that HBV replication and virion secretion can be significantly inhibited by Vps4 dominant negative, ATPase-defective, mutants K173Q and E228Q [62,63]. Similarly, wild-type Vps4, even at low dose DNA (0.5 µg), can inhibit HBV (*adr* and *ayw*) replication efficiently in transfected HepG2 cells, but not in HuH-7 cells. It appears that HepG2 and HuH-7 are cell lines with different contents of host factors for HBV replication [64].

### 7.2. ESCRT Machinery

It is known that ESCRT could mediate the export of a number of viruses. We investigated the potential effect of ESCRT on HBV replication and virion release in HepG2 cells, by using a siRNA knockdown screening of all the known ESCRT components. As shown in Table 1, we identified at least 15 ESCRT factors required for HBV replication. We focused our study on HGS (hepatocyte growth factor-regulated tyrosine kinase substrate) of ESCRT-0. Aberrant expression of HGS (over- or under-expression) can inhibit HBV replication. Unexpectedly, HGS could boost the release of naked capsids (subviral particles without envelope), while concurrently reduced the extracellular virions and HBsAg particles. Therefore, it appears that HGS exerted a strong positive effect on the secretion of naked capsids, at the expense of a reduced level of virions. HGS can associate with HBc in an ubiquitin-independent manner, and they are preferentially co-localized with each other near the cell periphery, instead of near the punctate endosomes in the cytoplasm. The secretion routes of HBV virions and naked capsids can be clearly distinguished based on the pleiotropic effect of HGS involved in the ESCRT-0 complex [61]. In summary, our work demonstrated the importance of an optimum level of HGS in HBV propagation. In addition to an effect on HBV transcription, HGS can diminish the pool size of intracellular nucleocapsids with ongoing genome maturation, probably in part by promoting the secretion of naked capsids. Recently, HGS was also found to be important in the life cycle of RNA viruses, such as chikungunya virus [65] and HTLV-2 [66]. 

In addition to HGS of ESCRT-0, other ESCRT components had also been found to be involved in HBV virion morphogenesis. For example, RNAi-induced depletion of the ESCRT-II components EAP20, EAP30 and EAP45, reduced pregenomic RNA encapsidation and virion secretion (Table 1) [61,67]. Similarly, coexpression of HBV and mutated ESCRT-III components CHMP3, CHMP4B, or CHMP4C, blocked HBV assembly and egress [63]. Like CHMP and Vps4 mutants, excessive amount of gamma 2-adaptin also blocked HBV production. Altogether, these results demonstrate that HBV exploits the ESCRT machinery for virion morphogenesis and export. For a review focusing more on the host cell machineries involved in HBV maturation, morphogenesis and egress, such as ESCRT, Rab, and COPII, the readers are advised to consult the previous [68] and the current article in this series by Dr. Prange [69].

### 7.3. Cellular Kinase and HBc Phosphorylation in Virion Secretion 

HBV-associated protein kinase activity was first detected back in the early 80s [70,71]. Pugh et al. reported that alkaline phosphatase treatment could remove the SDS-PAGE heterogeneity of the major duck hepatitis B virus (DHBV) core particle proteins in the cytoplasm [72]. In contrast, core protein from the serum of infected animals displayed no heterogeneity. Therefore, core protein dephosphorylation could occur before virion secretion. This correlation between capsid protein dephosphorylation and virion secretion was later confirmed by using mass spectrometry analyses of DHBV nucleocapsids [73]. Because phospho-mimetic substitutions from serine to aspartic acid in the DHBV core protein did not block the secretion of DNA-containing virions [74], it was concluded that DHBV core protein dephosphorylation may not be essential for the subsequent core-envelope interaction and virion secretion. 

As will be discussed here, it is a complicated issue whether this correlation between capsid dephosphorylation and virion secretion reflects a cause-effect relationship between the two. Does capsid phosphorylation or dephosphorylation contribute to the efficiency of genome-containing virion secretion? A few confounding factors are as outlined below: First, in addition to virion secretion, HBc phosphorylation/dephosphorylation is known to be critical in several earlier events in HBV life cycle, such as capsid assembly, capsid stability, RNA encapsidation, DNA synthesis and nucleic acid chaperone activity [41,49,75,76,77,78]. Similarly, multiple functions of capsid protein phosphorylation in DHBV replication had been reported. For example, the lack of phosphoserine at the residue 257 and at residues 257 and 259 stimulates covalently closed circular DNA synthesis (ccc DNA) and virus production, respectively [79]. Another DHBV core mutant S245D is also known for its enhanced capsid trafficking and genome release into the nucleus [80]. Culture supernatants from mutant S245D, but not S245N, is infectious (with reduced efficiency) in primary duck hepatocytes [80]. This result suggests that virion secretion is not significantly affected by S245D. Altogether, it is always difficult for a genetic approach to affecting only phosphorylation and virion secretion, without any other concurrent pleiotropic effects on earlier events preceding virion secretion. Second, for human HBV, the cytoplasmic tail of HBc has an arginine-rich domain (ARD), which contains closely clustering residues of 7 serine and one threonine for potential phosphorylation and dephosphorylation, as analyzed by biochemistry assays [41,49,81]. Similarly, DHBV capsid protein contains three serines and one threonine in the C-terminal domain. These potential phosphate acceptor sites are adjacent to a proline residue, suggesting an SP or TP motif for capsid protein phosphorylation [82]. Six additional serines are not adjacent to a proline residue, and thus while they may still serve as a phosphate acceptor site, they cannot be a substrate for an SP-specific kinase. In theory, a large number of differentially phosphorylated capsid protein patterns can be generated by various selective combinations of site-specific phosphorylation or dephosphorylation. It is technically challenging to test and compare quantitatively all the potential patterns of capsid protein phosphorylation. Ultimately, it will be informative to know if a particular HBc or DHBc genetic mutant with a specific phosphorylation-mimicking pattern, does or does not exist in physiological conditions in culture or in nature. Third, this issue is further complicated by the presence of hundreds of cellular kinases and phosphatases in hepatocytes. Members of the same kinase family often exhibited functional redundancy. To date, many different kinases and phosphatases have been studied in HBV biology, including protein kinase C [83,84], SRPK1/2 [41,81,85], CDK2 [86], PLK1 [87] and protein phosphatase 1 [88]. In one of these reports, inhibition of protein kinase C phosphorylation of HBV capsids resulted in the inhibition of virion formation and increased intracellular capsid accumulation [84]. Considering such a complex nature in HBc phosphorylation, it remains to be further investigated in dissecting the relationship between HBc phosphorylation or dephosphorylation and virion secretion.

### 7.4. Host Restriction Factor in Virion Secretion

BST-2/tetherin is an interferon-inducible antiviral cellular protein. It is known that BST-2 can block the egress of various enveloped viruses, including HIV-1 and HBV. Depending on the host cell lines, HBV virion secretion can be regulated by BST-2 in a cell line-dependent manner. For example, BST-2 restriction for HBV virion secretion is evident in HepG2 cells, but not apparent in HuH-7 cells. HBV can evade BST-2 restriction by the physical interaction between HBsAg and BST-2. By microscopy, BST-2 is co-localized with HBsAg at multivesicular bodies (MVBs) [89]. The expression of a BST-2 mutant truncated with the HBsAg-binding domain promoted a strong restriction on HBV virion secretion [90]. In another study, full-length BST-2, but not the C-terminal glycosyl-phosphatidylinositol (GPI) anchor-truncated form, inhibited HBV virion egress from HepAD38 cells [91]. Microscopy analyses demonstrated that the tethering of HBV virions occurs in the intracellular cisterna and that BST-2 is colocalized with HBV virions on the multivesicular body, which is the HBV virion budding site. In a PEG-containing in vitro infection system, two interferon-stimulated genes (ISGs), ISG20 and BST-2, restricted HBV spread in NTCP-expressing hepatoma cells [92]. Overall, the BST-2-HBsAg interaction could be a potential anti-viral drug target.

## 8. Persistence and Genome Maturity

**Figure 10:**
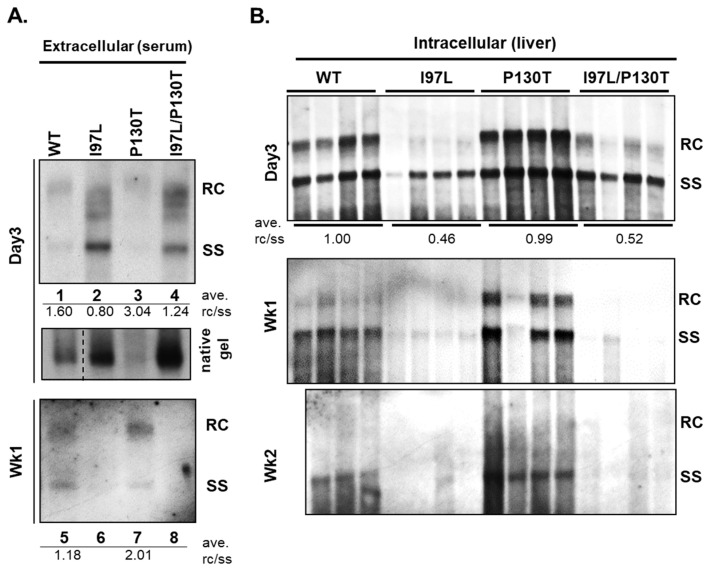
Rapid decline of HBV DNA containing an I97L mutation in a hydrodynamic injection mouse model. In contrast, genome hypermaturation of HBc mutant P130T is correlated with prolonged persistence of viral DNA. Both extracellular and intracellular viral DNAs were compared among four different groups of BALB/c mice, hydrodynamically injected with plasmid DNAs of WT, mutant I97L, mutant P130T, and double mutant I97L/P130T, respectively. (**A**) Extracellular virion-associated HBV DNAs were prepared from pooled mouse sera of the same experimental group at 3 and 7 dpi, followed by Southern blot analysis. The rc/ss ratio reflects the degree of HBV genome maturity. Single mutant P130T exhibited a higher degree of genome maturity than WT and other mutants. The mutation P130T can only partially upshift the immature SS DNA of mutant I97L to the higher molecular weight RC form at 3 dpi (lane 2 vs. lane 4). (**B**) Intracellular core-associated HBV DNA was extracted from liver tissues at 3 days, 1 week and 2 weeks post-injection before Southern blot analysis. Mutant P130T exhibited a stronger signal of full-length RC form than WT and mutant I97L. Unexpectedly, HBV DNA of mutant P130T is more persistent than WT and mutant I97L at 1 and 2 weeks post-injection. The mutation P130T could rescue neither the deficiency in RC DNA nor the poor persistence of mutant I97L. (with permission from Wu et al., 2019) [23].

In addition to the cell culture system, we investigated immature secretion in the hydrodynamic mouse model. Consistent with the previous results based on cell culture and transfection [17], mutant I97L in mice exhibited pleiotropic phenotypes. Extracellularly, the serum samples contained an excessive amount of HBV virions with immature genomes (left panel, Figure 10) [23]. Intracellularly, the liver samples presented significantly reduced amounts of intracellular RC and SS DNAs (Figure 10) [23]. This intracellular deficiency in viral DNA synthesis of mutant I97L in mice is more consistent with the results of mutant F97L in HuH-7 cells [17], and less consistent with the result of mutant I97L in HepG2 cells [18]. It is likely that the reduced intracellular DNA signal of mutant I97L in HepG2, was obscured in part by the high dose DNA at the plateau level (7–10 μg plasmid DNA) by calcium-phosphate transfection, and in part by the over-exposed autoradiogram. Using a lower dose amount of DNA (1–2 μg) in liposome-mediated transfection, we also observed reduced intracellular viral DNA replication of mutant I97L in HepG2 cell culture (Szu-Yao Wu and Chiaho Shih, unpublished results). Overall, these pleiotropic phenotypes in vivo were observed in both immune-competent and immunodeficient mice [23]. Interestingly, an unexpected phenotype of mutant I97L in the mouse model is the less persistent intrahepatic and serum HBV DNAs than the wild type HBV (Figure 10).

As mentioned earlier, mutant P130T, by itself, exhibited an intracellular phenotype of genome hypermaturation with enriched amount of near full-length RC DNA. In addition, in the context of the double mutant F97L/P130T, the nonselective immature secretion of mutant F97L was lost, and virion-associated HBV DNA was back to a WT-like pattern with predominant mature genome [43]. We asked whether the genome hypermaturation of mutant P130T can be faithfully reproduced in the BALB/c mice, and whether its compensatory effect on immature secretion can also be observed in vivo. Although mutant P130T displayed a hypermaturation phenotype in vivo, it cannot efficiently rescue the immature virion secretion of mutant I97L (Figure 10). Nevertheless, the mutation P130T exhibited in vivo a novel phenotype in prolonging the persistence of HBV genome in serum and hepatocytes at wk 1 or wk 2 post-injection (Figure 10). Therefore, there is a correlation between genome maturation and HBV DNA persistence (Table 2). In summary, we validated the cell culture-based phenotypes using a hydrodynamic mouse model. Unexpectedly, we found genome maturity favors HBV persistence in vivo.

## 9. Conclusions

To understand better the biological significance of frequent naturally occurring HBV core antigen variants, we identified an immature secretion phenotype of mutant 97L. The pleiotropic phenotypes of this mutant 97L were dissected by using a number of approaches, including a cis-trans genetic complementation test and hydrodynamic delivery mouse models. Compensatory mutations at HBV core or envelope can erase the immature secretion of mutant 97L. Kinetic analysis of virion secretion in a time-course experiment revealed no significant difference in the release of immature or mature genomes between wild type HBV and mutant 97L. A hydrophobic pocket around the core amino acid 97 could play a role in relaying the genome maturation signal from capsid interior to the surface of nucleocapsids undergoing genome maturation. ESCRT is an important cellular machinery for HBV trafficking and virion secretion. A common theme of naturally occurring core variants is their somewhat loosened coupling in the tempos between genome maturation and virion secretion. 

Using a hydrodynamic mouse model, we observed rapid decline of HBV DNA of mutant I97L as well as a correlation between genome hypermaturation and in vivo persistence of HBV DNA of mutant P130T (Figure 10). Can these mouse results be extended to human patients? It is known that the serum HBV DNA level is a major predictor of spontaneous seroclearance of HBeAg, HBV DNA and HBsAg [93,94]. Therefore, it is logical to predict a higher spontaneous clearance rate for chronic carriers containing an HBc 97L mutation in longitudinal follow up studies. Indeed, in a small cohort study on chronic hepatitis B patients, core mutation I97L appeared to be useful in predicting a persistent low level of HBV DNA, normal ALT, and HBsAg clearance [95]. Quantitative HBsAg monitoring in the late phase of chronic infection could provide us a better understanding and management of HBeAg-negative chronic carriers [96]. It is tempting to speculate here that mutation I97L might be used in combination with HBsAg quantification to achieve an even higher predictive power for functional cure (loss of HBsAg). Furthermore, while NUC (nucleos(t)ide analogs) therapy can suppress serum HBV DNA to an undetectable level, continuous life-long NUC therapy is needed to prevent HBV from relapse [5,6,7,97,98,99,100]. It will be interesting to compare the efficacies of finite or indefinite life-long NUC therapy between chronic HBeAg-negative patients with versus without an I97L or P130T mutation. 

Another issue remaining to be resolved is whether these immature virions of mutant 97L are infectious by in vivo or in vitro infection assays. Mutant 97L secretes virions containing almost equal amounts of mature and immature genomes. By density gradient ultracentrifugation, we were not able to separate immature from mature virion populations. The infectivity issue of 97L virions can be better addressed, if the technical challenge in virion purification can be circumvented. 

Overall, these HBV variants offered us a useful research tool for our investigation on the mechanism of virion release, which could serve as a target for drug discovery in the near future. It is also very likely that mutant 97L could predict NUC therapeutic efficacy or a higher spontaneous clearance rate of serum HBV DNA and HBsAg in chronic hepatitis B patients. 

## Figures and Tables

**Table 1:** Tested ESCRT factors that affected HBV replication in HepG2 cells.

Complex	Suppression of HBV DNA Replication
ESCRT-0	si-HGS
	si-STAM1, 2
ESCRT-I	si-VPS28
	si-VPS37B *
	si-UBAP1
ESCRT-II	si-EAP20
	si-EAP45
ESCRT-III & related factors	si-CHMP4A, B *
	si-CHMP3
	si-CHMP2A, B
	si-CHMP1A, B
	si-IST1
VPS4 complex	si-VPS4A
	si-VTA1 *

* The effects of VPS37B, CHMP4B and VTA1 on HBV replication are modest, yet reproducible. (with permission from Chou et al., 2015) [61].

**Table 2:** Correlation between viral DNA persistence and genome maturation.

HBc Variants	Genome Maturity *	Persistence ^#^
I97L	+	+
WT (*adr*)	++	++
P130T	+++	+++

* + immature genome, ++ mature genome, +++ hypermature genome. ^#^ intrahepatic RC DNA detectable at post-injection: + 3 days, ++ 1 wk, +++ 2 wks (from Wu et al., 2019) [23].

## Abbreviations

ARD	arginine-rich domain
BST-2	Bone marrow stromal antigen 2, also known as tetherin
cccDNA	covalently closed circular DNA
CDK2	cyclin-dependent kinase 2
CLP	capsid-like particles
CTL	cytotoxic T-lymphocytes
DHBc	DHBV core (capsid) protein
DHBV	duck hepatitis B virus
DS DNA	double-stranded DNA
ESCRT	The Endosomal Sorting Complex Required for Transport
GPI	glycosylphosphatidylinositol
HBc	HBV core protein
HBeAg	HBV e antigen
HBsAg	HBV surface antigen
HBV	human hepatitis B virus
HGS	hepatocyte growth factor-regulated tyrosine kinase substrate
HTLV-2	Human T Cell Leukemia Virus Type 2
ISG	interferon-stimulated genes
NTCP	sodium taurocholate cotransporting polypeptide
PEG	polyethylene glycol
PKA	cyclic AMP dependent protein kinase A
PKC	Ca2+ and/or lipid-activated protein kinase C
PLK1	polo-like kinase 1
RC DNA	relaxed circle DNA
SGHBV	Snow Goose hepatitis B virus
SKO	HBsAg protein synthesis knockout (SKO)
SRPK1 and 2	serine/arginine-rich protein kinase 1 and 2
SS DNA	single-stranded DNA
WHV	woodchuck hepatitis B virus
WT	wild type
